# Evidence of SARS-CoV-2 RNA in an Oropharyngeal Swab Specimen, Milan, Italy, Early December 2019

**DOI:** 10.3201/eid2702.204632

**Published:** 2021-02

**Authors:** Antonella Amendola, Silvia Bianchi, Maria Gori, Daniela Colzani, Marta Canuti, Elisa Borghi, Mario C. Raviglione, Gian Vincenzo Zuccotti, Elisabetta Tanzi

**Affiliations:** University of Milan, Milan, Italy (A. Amendola, S. Bianchi, M. Gori, D. Colzani, E. Borghi, M.C. Raviglione, G.V. Zuccotti, E. Tanzi);; Memorial University of Newfoundland, St. John’s, Newfoundland, Canada (M. Canuti)

**Keywords:** COVID-19, coronavirus disease, SARS-CoV-2, severe acute respiratory syndrome coronavirus 2, viruses, respiratory infections, zoonoses, SARS-CoV-2 RNA, oropharyngeal swab, skin manifestations, Milan, Italy

## Abstract

We identified severe acute respiratory syndrome coronavirus 2 RNA in an oropharyngeal swab specimen collected from a child with suspected measles in early December 2019, ≈3 months before the first identified coronavirus disease case in Italy. This finding expands our knowledge on timing and mapping of novel coronavirus transmission pathways.

Coronavirus disease (COVID-19) symptoms can encompass a Kawasaki disease–like multisystem inflammatory syndrome and skin manifestations that accompany common viral infections such as chickenpox and measles (*1*,*2*). Some of the earliest reports of COVID-19 cutaneous manifestations came from dermatologists in Italy. In fact, Italy was the first Western country severely hit by the COVID-19 epidemic. The first known COVID-19 case in Italy was reported in the town of Codogno in the Lombardy region on February 21, 2020. However, some evidence suggests that severe acute respiratory syndrome coronavirus 2 (SARS-CoV-2) had been circulating unnoticed for several weeks in Lombardy before the first official detection (*3*). Phylogenetic studies highlighted an early circulation of SARS-CoV-2 in Italy and suggest multiple introductions of the virus from China and Germany, followed by an autochthonous transmission (*4*,*5*). Furthermore, environmental surveillance has unequivocally demonstrated the presence of the virus, at concentrations comparable to those obtained from samples collected at later stages of the pandemic, in the untreated wastewater of the Milan area as early as mid-December 2019 (*6*).

As participants in Italy’s Measles and Rubella Network, a sensitive case-based surveillance system, we observed in Milan during late autumn 2019 cases of suspected measles in patients who eventually tested negative for measles. We therefore retrospectively explored a possible etiologic involvement of SARS-CoV-2 in these non–measles-linked rash cases.

We analyzed oropharyngeal swabs specimens collected during September 2019–February 2020 from 39 consenting patients (mean age 19.9 years [range 8 months–73 years]). All laboratory procedures were conducted in a university research laboratory, accredited according to World Health Organization standards, dedicated exclusively to the surveillance of measles and rubella, and therefore designated as free from SARS-CoV-2. RNA strands stored at −80°C were tested by an in-house heminested reverse transcription PCR assay for the amplification of a 470-bp fragment of the gene encoding the SARS-CoV-2 spike protein. Primers used during the first amplification step were Out_f 5′-AGGCTGCGTTATAGCTTGGA-3′ and MaSi_Ar 5′-ACACTGACACCACCAAAAGAAC-3′. Primers used for the second step were SiMa_Bf 5′-TCTTGATTCTAAGGTTGGTGGT-3′ and MaSi Ar 5′-ACACTGACACCACCAAAAGAAC-3′. Positive and negative controls also were included in each PCR test and performed as expected.

One oropharyngeal swab specimen tested positive. The amplicon was sequenced by using Sanger technology, resulting in a sequence of 409 bp. Sequence analysis performed by using BLAST (https://blast.ncbi.nlm.nih.gov/Blast.cgi) showed 100% identity to the reference sequence Wuhan-HU-1 (GenBank accession no. NC_045512.2) as well as to sequences of other SARS-CoV-2 strains circulating worldwide at a later stage; therefore, accurately determining the origin of the identified strain was not possible. The specimen was confirmed as positive by repeated amplification and sequencing, and all other specimens were repeatedly negative. The sequence (SARS-CoV-2_Milan_Dec2019 [GenBank accession no. MW303957]) was identified in a specimen collected from a 4-year-old boy who lived in the surrounding area of Milan and had no reported travel history. On November 21, the child had cough and rhinitis; about a week later (November 30), he was taken to the emergency department with respiratory symptoms and vomiting. On December 1, he had onset of a measles-like rash; on December 5 (14 days after symptom onset), the oropharyngeal swab specimen was obtained for clinical diagnosis of suspected measles. This patient’s clinical course, which included late skin manifestations, resembles what has been reported by other authors; maculopapular lesions have been among the most prevalent cutaneous manifestations observed during the COVID-19 pandemic, and several studies have noticed a later onset in younger patients (*7*).

We describe the earliest evidence of SARS-CoV-2 RNA in a patient in Italy, ≈3 months before Italy’s first reported COVID-19 case. These findings, in agreement with other evidence of early COVID-19 spread in Europe, advance the beginning of the outbreak to late autumn 2019 (*6*,*8*–*10*). However, earlier strains also might have been occasionally imported to Italy and other countries in Europe during this period, manifesting with sporadic cases or small self-limiting clusters. These importations could have been different from the strain that became widespread in Italy during the first months of 2020. Unfortunately, the swab specimen, which was collected for measles diagnosis, was not optimal for SARS-CoV-2 detection because it was an oropharyngeal rather than a nasopharyngeal swab specimen and it was collected 14 days after the onset of symptoms, when viral shedding is reduced. In addition, thawing might have partially degraded the RNA, preventing the sequencing of longer genomic regions that could have been helpful in determining the origin of the strain.

This finding is of epidemiologic importance because it expands our knowledge on timing and mapping of the SARS-CoV-2 transmission pathways. Long-term, unrecognized spread of SARS-CoV-2 in northern Italy would help explain, at least in part, the devastating impact and rapid course of the first wave of COVID-19 in Lombardy. Full exploitation of existing virologic surveillance systems to promptly identify emerging pathogens is therefore a priority to more precisely clarify the course of outbreaks in a population. Further studies aimed at detecting SARS-CoV-2 RNA in archived samples suitable for whole-genome sequencing will be crucial at determining exactly the timeline of the COVID-19 epidemic in Italy and will be helpful for the preparedness against future epidemics.
